# Rapid Progression of Coronary Atherosclerosis: A Review

**DOI:** 10.1155/2015/634983

**Published:** 2015-12-28

**Authors:** Priyank Shah, Sharad Bajaj, Hartaj Virk, Mahesh Bikkina, Fayez Shamoon

**Affiliations:** Department of Cardiology, 703 Main Street, Paterson, NJ 07503, USA

## Abstract

Atherosclerosis is chronic disease, the prevalence of which has increased steadily as the population ages. Vascular injury is believed to be critical initiating event in pathogenesis of spontaneous atherosclerosis. Syndrome of accelerated atherosclerosis has been classically described in patients undergoing heart transplantation, coronary artery bypass graft, and percutaneous transluminal coronary angioplasty. In contrast to spontaneous atherosclerosis, denuding endothelial injury followed by thrombus formation and initial predominant smooth muscle cell proliferation is believed to be playing a significant role in accelerated atherosclerosis. There is no universal definition of rapid progression of atherosclerosis. However most studies describing the phenomenon have used the following definition: (i) > or = 10% diameter reduction of at least one preexisting stenosis > or = 50%, (ii) > or = 30% diameter reduction of a preexisting stenosis <50%, and (iii) progression of a lesion to total occlusion within few months. Recent studies have described the role of coronary vasospasm, human immunodeficiency virus, various inflammatory markers, and some genetic mutations as predictors of rapid progression of atherosclerosis. As research in the field of vascular biology continues, more factors are likely to be implicated in the pathogenesis of rapid progression of atherosclerosis.

## 1. Introduction

The prevalence of atherosclerosis has increased steadily due to aging population. Economic development and urbanization have promoted habits of diet rich in saturated fat and diminished physical activity, which favors atherosclerosis [1]. Traditionally two types of atherosclerosis were described, spontaneous and accelerated [2]. Accelerated atherosclerosis mainly occurs in patients after heart transplant, coronary artery bypass graft (CABG), and percutaneous transluminal coronary angioplasty (PTCA) [2]. Although atherosclerosis is believed to progress over many years, it has been increasingly noted to progress over few months to 2-3 years in few patients without traditional factors for accelerated atherosclerosis. Hence the term rapid progression of atherosclerosis has been used in recent years.

Vascular injury in the form of endothelial injury is a critical initiating event in pathogenesis of both spontaneous and accelerated atherosclerosis [2]. In spontaneous atherosclerosis, there is chronic damage to arterial endothelium by turbulence of blood flow or other injuries, which leads to nondenuding functional alterations of endothelial cells (type 1 injury). This leads to lipid accumulation, the initial predominant feature in this type. Adhesion of monocytes and platelets either simultaneously or at a later time occurs. Along with altered endothelium, these cells release various growth factors, leading to migration and proliferation of smooth muscle cells. This ultimately forms a typical atherosclerotic plaque [2]. In contrast to spontaneous atherosclerosis, accelerated atherosclerosis is initiated by significant denuding endothelial injury (type 2 or 3 injury). Once endothelium is denuded, immediate platelet aggregation and thrombus formation occur on subendothelium. Intact endothelium is a potent inhibitor of growth of smooth muscle cells. Hence endothelial denudation leads to early smooth muscle cell proliferation and fibrosis mediated by various factors released by platelets, leucocytes, and smooth muscle themselves. Accumulation of lipids occurs late in accelerated atherosclerosis [2].

## 2. Review

### 2.1. Accelerated Atherosclerosis

Accelerated atherosclerosis was initially described in the 1980s in patients undergoing heart transplant, CABG, and PTCA [2, 3]. Two main types of coronary lesions were described in transplant patients with coronary artery disease; the first type tends to be after the first postoperative year resembling spontaneous atherosclerosis and second type which occurs within first postoperative year characterized by diffuse concentric narrowing located primarily at distal secondary and tertiary vessels resembling accelerated atherosclerosis [3]. Type 2 endothelial injury (denudation of endothelium with damaged intima but intact internal elastic lamina and media) occurs in posttransplant patients. Mechanism of type 2 injury has been attributed to preimplantation injury, denervation, viral infection, hyperlipidemia, and immune injury [2]. A significantly higher percent of patients with premature allograft atherosclerosis have cytomegalovirus infection than those without premature coronary disease [4]. Although immune injury has been implicated in causing type 2 injury in transplanted hearts, immunosuppressive therapy has not been shown to reduce the incidence or severity of accelerated atherosclerosis in transplanted hearts suggesting multifactorial nature of the pathogenic process [5].

Coronary vein graft disease contributes to significant morbidity and mortality in post-CABG patients. About 20% vein grafts are occluded by 1 year and, at the end of 5 years, 35% vein grafts will be occluded [6–11]. Vein grafts are subject to mechanical injury during harvesting and handling intraoperative, leading to type 3 endothelial injury (denudation with damage to both intima and media). With exposure of vein graft to a relatively high pressure flow and presence of risk factors like hypercholesterolemia, a type 2 endothelial injury occurs early after CABG. This leads to thrombus formation and acute occlusion. Hence early vein graft occlusions are thrombotic in origin [2]. Moreover, arterialization of vein graft results in decrease in production of prostacyclin (platelet antiaggregator), increasing the thrombogenicity of vein grafts [12].

After PTCA, 5% have acute reocclusion and about 35% have restenosis within 6 months [2]. With balloon inflation there is type 2/3 endothelial injury leading to immediate platelet aggregation and thrombus formation. Platelet deposition is markedly reduced in 4 days, coinciding with partial regrowth of endothelium and periluminal cells. Subsequently, a process of intimal smooth muscle cell proliferation and reorganization of mural thrombus is evident in 10–14 days [2]. Since the advent of stents specifically drug eluting stents, accelerated atherosclerosis is not commonly seen after intervention in culprit vessel at the lesion site.

## 3. Rapid Progression of Atherosclerosis

Apart from the conditions described in accelerated atherosclerosis, coronary artery disease has been shown to progress significantly over period of months to few years in a subset of patients. Hence the term rapid progression of atherosclerosis has been used recently in the literature. Anatomical and physiological as well as inflammatory markers have been implicated in rapid progression of atherosclerosis [13–28]. There is no universal definition of rapid progression of atherosclerosis. However, most of the studies describing this phenomenon have used the following definition: (i) > or = 10% diameter reduction of at least one preexisting stenosis > or = 50%, (ii) > or = 30% diameter reduction of a preexisting stenosis <50%, and (iii) progression of a lesion to total occlusion over a few months [17–21, 23].

### 3.1. Role of Coronary Spasm

Nobuyoshi et al. in 1991 [16] suggested the role of spam in coronary disease progression. Although there were no predefined criteria to classify rapid progression in this study, most disease progression occurred within 4 years. A positive response to ergonovine provocation test was an independent predictor of disease progression in this study. Subsequently, a study on swine model showed occurrence of intramural hemorrhage with abrupt onset of coronary spasm. Intramural hemorrhage resulted in acute progression of coronary stenosis and sometimes resulted in persistent total coronary occlusion [15]. More recently there have been case reports of coronary spasm causing rapid progression of atherosclerosis over a period of few months to 3 years [13, 14].

Mechanisms by which spasm can lead to rapid progression of atherosclerosis are putative. It is believed that spasm can make the endothelium dysfunctional and can disrupt it [16]. Also the drag generated by spasm may mechanically tear the capillaries and this effect may be stronger with coronary spasm of abrupt than gradual onset. Moreover, spasm induced intramural hemorrhage might be an important factor responsible for acute progression of organic stenosis [15]. Coronary spasm can even lead to plaque rupture and coronary thrombosis [15]. Hence patients with vasospastic angina should be treated aggressively.

### 3.2. Role of Complex Stenosis Morphology

A complex coronary lesion is defined as those having irregular borders, overhanging edges, and intracoronary thrombi. Angiographic studies have indicated that complex lesion morphology is associated with increased risk of myocardial infarction [25]. Some studies have indicated that complex stenosis morphology is associated with rapid progression of atherosclerosis [25–28]. Kaski et al. studied 94 patients awaiting routine angioplasty. They showed significantly higher rates of rapid progression of coronary stenosis in complex lesions (22% versus 4%, *p* = 0.002). In their study, the definition of rapid progression was as follows: (i) > or = 20% reduction in minimal stenosis diameter of a preexisting lesion > or = 30% and (ii) progression of any lesion to a new total coronary occlusion. Time between two angiograms was 8 ± 3 months [28]. Although the use of aspirin was close to 100% in this study in both groups, there was no mention of statin use in either group. Another study showed significantly higher rates of rapid progression (defined as > or = 20% reduction in diameter) in complex lesions compared to smooth lesions (19% versus 5%, *p* = 0.003) [26]. The use of aspirin was about 72% and there was no mention regarding use of statins. These studies showing significantly higher rates of rapid progression in complex lesions were in the mid-1990s when statins were still not widely used. However, a recent study that evaluated the role of C-reactive protein (CRP) in rapid progression also showed significant difference in rates of rapid progression between complex and smooth lesions. In this study, although the use of statin was significantly lower in progressors versus nonprogressors (56% versus 77%, *p* = 0.008), after adjustment, presence of multiple complex lesions was an independent predictor of rapid progression of nonculprit lesions along with admission and postpercutaneous coronary intervention (PCI) CRP elevation [20].

The roughness of plaque surface is believed to cause increased thrombogenicity and platelet deposition. This then leads to cascade of events similar to that in accelerated atherosclerosis. Adherence of platelets to a disrupted intima followed by the release of procoagulant metabolites and concomitant enhanced vasoreactivity may predispose to thrombotic coronary occlusion [26].

### 3.3. Role of Inflammatory Markers

#### 3.3.1. Endothelin

Zouridakis et al. evaluated 224 coronary stenoses in 92 patients with chronic stable angina for rapid progression by 2 angiograms done 5.5 (±3) months apart. The definition of rapid progression was as follows: (i) > or = 10% diameter reduction of at least one preexisting stenosis > or = 50%, (ii) > or = 30% diameter reduction of a preexisting stenosis <50%, and (iii) progression of a lesion to total occlusion. They showed that plasma endothelin levels were significantly higher in patients with rapid progression than those without. But, the use of statins was very low (41% and 28% in nonrapid and rapid progressors, resp.). However, after adjustment of multiple factors, endothelin was an independent predictor of disease progression and endothelin levels >4.26 pg/mL were associated with 6-fold increase in risk of rapid disease progression [17]. Classic cardiovascular risk factors were not shown to be predictive of rapid progression of atherosclerosis in this study.

Endothelins are a family of potent vasoconstrictor peptides, which have cell growth-promoting and mitogenic properties. Macrophage-rich areas, hypercellular areas showing neovascularization, and areas with evidence of previous intraplaque hemorrhage were significantly associated with the presence of endothelin-1. Accelerated proliferation of vascular cells may be a contributing mechanism for rapid coronary lesion progression [17].

#### 3.3.2. Lipoprotein(a) (Lp(a))

Terres et al. studied 79 patients with at least one coronary artery stenosis > or = 50%. Coronary angiography was performed at a mean of 66 (±25) days. Rapid progression was defined as follows: (i) an increase in >10% in stenosis severity in at least one stenosis > or = 50%, (ii) occurrence of new stenosis > or = 50%, or (iii) occlusion of a formerly patent vessel. They found that elevated serum Lp(a) levels were independently associated with rapid disease progression [18]. Although other makers of inflammation were not evaluated in this study, the classic cardiovascular risk factors did not differ in the group that progressed rapidly versus the group that did not.

Lp(a) is a plasma lipoprotein composed of apolipoprotein B-100 and shares a high degree of structural homology with plasminogen. It attenuates the activation of plasminogen by streptokinase and tissue plasminogen activator and there is competition between Lp(a) and plasminogen for binding sites on endothelial cells, platelets, and fibrinogen and fibrin. Thus it exerts antifibrinolytic action. Lp(a) is also shown to stimulate the proliferation of human smooth muscle cells [18].

#### 3.3.3. CRP

Nakachi et al. studied 153 patients with non-ST-elevation acute coronary syndrome who underwent PCI and follow-up coronary angiography in 7 months. Rapid progression was defined as follows: (i) > or = 10% diameter reduction of at least one preexisting stenosis > or = 50%, (ii) > or = 30% diameter reduction of a preexisting stenosis <50%, and (iii) progression of a lesion to total occlusion. Admission CRP elevation (odds ratio 2.92) and post-PCI (48 hours) CRP elevation (odds ratio 2.67) along with multiple complex lesions were independent predictors of rapid progression of nonculprit lesions. This study did not evaluate other inflammatory markers. Patients with conditions known to elevate CRP were excluded from the study [20]. Another study of 124 chronic stable angina patients on waiting list for angioplasty who underwent repeat study after a mean time of 4.8 months showed CRP as an independent predictor of rapid progression [19]. Other markers associated with rapid progression in this study were neopterin, matrix metalloproteinase-9 (MMP-9), and soluble intercellular adhesion molecule-1 (ICAM-1). Rapid progression was defined as the same way as described earlier in the paragraph.

Few things can explain this association. Trauma associated with diagnostic part of the procedure may trigger an inflammatory response. Rise of CRP after PCI can be attributed to inflammatory stimuli associated with plaque disruption and coronary artery injury during balloon inflation and stent implantation. Disruption of vulnerable plaque also causes microembolization, resulting in low-grade myonecrosis, which has been suggested to be the main source of inflammatory stimuli in such patients. It is believed that CRP is produced endogenously in the vessel wall or in coronary atherosclerotic plaques injured by PCI. Rapid progression of nonculprit lesions can result from negative remodeling and organization of plaque hemorrhages or from repeated subclinical cycles of rupture, hemorrhage, and organization [20]. The magnitude of acute phase response and hence the elevation of CRP is determined by individual responsiveness. Hence, those patients with heightened general inflammatory response will have higher elevation of CRP after PCI and thus are more likely to have rapid progression of atherosclerosis.

#### 3.3.4. Neopterin/MMP-9/ICAM-1

Neopterin, MMP-9, and ICAM-1 were shown to independently predict rapid progression along with CRP levels in a study of 124 chronic stable angina patients [19]. MMPs are produced by activated macrophages crucial in the process that leads to disruption of fibrous cap of atheromatous lesions. Neopterin is secreted by activated macrophages on stimulation by interferon-gamma. These results suggest that monocyte/macrophage activation plays a pathogenic role in plaque vulnerability and accelerated stenosis progression. Inflammatory markers were assessed only at one point in time, so dynamic changes in inflammatory markers during follow-up could not be ascertained in this study.

#### 3.3.5. Osteopontin

Mazzone et al. studied 77 patients with acute coronary syndrome (ACS) and chronic angina who had coronary angiograms done 6 months apart. Baseline osteopontin levels were associated with rapid coronary plaque progression [21]. Rapid progression was defined as (i) > or = 10% diameter reduction of at least one preexisting stenosis > or = 50%, (ii) > or = 30% diameter reduction of a preexisting stenosis <50%, (iii) progression of a lesion to total occlusion, or (iv) diameter reduction of 50% or more in previously successfully dilated lesion. Other markers measured in this study were CRP and interleukin-6, which were not statistically significant after adjustment of other variables.

Osteopontin in the plaque is believed to exert its effect through upregulation within and in proximity to activated cells, thereby becoming responsible for changes leading to instability, and inducing matrix metalloproteinase release, angiogenesis, hemorrhage, fibrous cap degradation, and thrombotic complications [21]. The reason for nonsignificant difference between CRP levels may have been due to small study sample.

#### 3.3.6. Lipoprotein-Associated Phospholipase A2 (Lp-PLA2)

A study of 123 patients with ACS who underwent PCI and follow up (mean interval of 1 year) angiography showed Lp-PLA2 to be an independent predictor of rapid progression of nonculprit coronary lesions. Rapid progression was defined as (i) > or = 10% diameter reduction of at least one preexisting stenosis > or = 50%, (ii) > or = 30% diameter reduction of a preexisting stenosis <50%, and (iii) progression of a lesion to total occlusion [23]. Measurement of the marker was done only one time (12–14 hours after PCI).

Lp-PLA2 is a novel inflammatory marker involved in bioactive lipid formation and pathogenesis of atherosclerosis. It is present in thin cap fibroatheromas and is preferentially secreted by macrophage and hydrolysed chemoattractants could contribute to development and progression of atherosclerotic lesions.

### 3.4. Role of Spotty Calcification

Spotty calcification is defined as presence of lesions 1–4 mm in length containing an arc of calcification <90 degrees. A total of 1347 stable patients who underwent serial evaluation with intravascular ultrasound imaging after a mean follow-up of about 650 days in 6 trials were studied. Spotty calcification was associated with greater progression of percent atheroma volume (PAV) [22]. After adjustment for baseline PAV, age, gender, diabetes mellitus, baseline LDL and HDL cholesterol levels, and baseline statin use, the results remained significant. Although intensive low-density lipoprotein cholesterol and blood pressure lowering therapy slowed disease progression, these efficacies were attenuated in patients with spotty calcification.

Coronary artery calcification can occur via a complex, regulated process of biomineralization resembling osteogenesis. Spotty calcified plaque may contain more lipid and inflammatory materials than noncalcified lesions and therefore is likely to undergo greater atheroma progression.

### 3.5. Role of Systemic Diseases

#### 3.5.1. Rheumatoid Arthritis

Rheumatoid arthritis (RA) is associated with increased risk of cardiovascular events and the mortality is high [29]. Although no study has specifically looked at progression of coronary atherosclerosis in RA patients, del Rincón et al. showed rapid progression of atherosclerosis in carotid arteries in RA patients [24]. In this study of 487 RA patients, elevated ESR was independently associated with rapid progression of atherosclerosis over a period of 2.8 years as assessed by carotid intima media thickness. Rapid progression of atherosclerosis in these patients most likely seems to be related to inflammation. Studies have shown decreased progression of atherosclerosis with treatment with methotrexate or biologic agents [24, 29, 30].

### 3.6. Role of Chronic Cocaine Use

No studies have evaluated the effect of chronic cocaine use in progression of atherosclerosis. However, 2 cases have been reported whereby significant progression of coronary atherosclerosis occurred in young patients (in their 30s) over a period of 9–16 months [31]. Both patients used crack cocaine on chronic basis. Chronic cocaine use could produce intermittent increases in shear forces on the coronary vessels and thereby promote atherosclerosis. It also leads to endothelial damage, increased mast cells, and increased platelet aggregation all of which likely play a role in rapid progression of atherosclerosis [31].

## 4. Conclusion

Syndrome of accelerated atherosclerosis had been classically described in patients after heart transplant, CABG, and PTCA. However, rapid progression of atherosclerosis seems to occur in certain other groups of patients with vasospasm, complex stenosis morphology, certain elevated inflammatory markers, spotty calcification, systemic disease like RA, and chronic cocaine use. Increased inflammation is probably the final pathway that leads to rapid progression of atherosclerosis. As far as role of inflammatory markers is concerned, most of the studies focused on one marker. So it remains to be seen if all the markers are simultaneously studied, which ones will predict the rapid progression of atherosclerosis. A recent study showed critical impairment of adaptive immune response in patients with ACS with reduced expression of regulatory T cells and enhanced effector T cell responsiveness [32]. Whether these impairments lead to enhanced inflammatory response leading to rapid progression of atherosclerosis is unknown. Future studies should study such immunologic markers along with inflammatory markers. Progression of atherosclerosis was determined using coronary angiography in most of these studies. However, intravascular ultrasound (IVUS) and optical coherence tomography (OCT) are more sensitive in detecting plaques [33, 34]. IVUS and OCT can provide much more information about the process going on in the vessel wall and about the plaque morphology as well. It is possible that coronary angiography which is merely a luminogram may have underestimated progression of atherosclerosis in previously described studies. Although it is not routinely acceptable to use IVUS or OCT to assess progression of atherosclerosis, it would provide much more insight if a study could assess progression of atherosclerosis in major epicardial vessels using these imaging modalities. Since none of the above studies were done in a randomized controlled manner, future randomized studies in this subset of patients will prove to be extremely helpful in risk stratification and management of such patients. As far as clinicians are concerned, this subset of patients should be followed and treated aggressively.
